# Palliative care and vascular surgery: status quo, needs, barriers and current challenges from the perspective of vascular surgery specialists

**DOI:** 10.1186/s12904-026-02113-0

**Published:** 2026-04-24

**Authors:** Michael Rechenmacher, Annette Schnell, Martina Schulz, Wolfgang Herr, Wilma Schierling, Karin Pfister, Ruth Mair

**Affiliations:** 1 Clinic for Internal Medicine III, University Hospital Regensburg, Regensburg, Germany; 2 Center for Palliative Care, University Hospital Regensburg, Regensburg, Germany; 3 Department of Vascular Surgery, University Hospital Regensburg, Regensburg, Germany

**Keywords:** palliative care, end of life care, vascular surgery, vascular medicine, early integration, barriers, palliative symptom management, relatives

## Abstract

**Background:**

Palliative care is a concept for all patients suffering from life threatening diseases. It is not limited to cancer patients or to the last few days of life. Current research on diseases and concomitant diagnoses in vascular surgery suggests a high need for palliative care. Specific palliative concepts for vascular medicine are still lacking.

**Methods:**

We conducted a study on the subjective views of experts in vascular surgery in Germany using a qualitative interview method to examine various aspects of palliative care in vascular surgery.

**Results:**

A total of 609 relevant text passages were identified and coded into seven main categories and 43 subcategories. The coding categories were as follows: description of the current situation of care in vascular surgery, identification of palliative vascular surgery patients’, description of palliative care symptoms requiring treatment, methods for identifying palliative vascular surgery patients, desired effects of palliative care interventions, barriers to implementation of palliative care, and the role and needs of relatives.

**Conclusions:**

The results of this study suggest that vascular surgery palliative patients may represent a specific group of patients, necessitating the development of a customised palliative care concept to the specific needs of this group. The specific requirements for the care of these patients and numerous barriers currently prevent adequate palliative care of these patients. The results of our study, which we were able to extract from interviews with experienced vascular surgeons and their insights into everyday clinical practice, confirm the conclusions drawn in published clinical studies and supplement them with further aspects, which together appear necessary for improved care of this patient group in the future. Central aspects to be considered include suitable training of vascular surgeons for a comprehensive understanding of palliative care, as well as advance care planning and written standards defining the situations in which palliative care should routinely be offered.

## Background

Palliative care is a concept for all patients suffering from life threatening diseases. It is not limited to providing the best possible care during the last few days of life. Rather, it focuses on holistic care for the ill person and their relatives, starting from the diagnosis of a life-threatening disease [1]. To date, close integration of palliative care is an accepted standard in oncology [2]. Beyond oncology, the integration of palliative care is often only rudimentary or is still being developed [3]. Structured research regarding the relationship between vascular surgery and palliative care, the extent and type of vascular patients’ distress, the need for palliative care support, or gaps in care for patients and their relatives has rarely been published. The available data on these topics show an inadequate provision of palliative care in this patient group [4]. However, there is definite evidence that the integration of palliative care may be beneficial for palliative patients with vascular diseases [5].

## Methods

### Design

This qualitative interview study examined the current status of and challenges facing the clinical care of palliative patients of vascular surgery from the perspective of experienced vascular surgeons in Germany. Therefore, the semi-structured interview guide consisted of open questions that encouraged experts to describe different facets important to palliative care: (a) the interface between vascular surgery and palliative care, (b) which forms of care, basic or supplementary specialized palliative care, are considered necessary and helpful in various clinical situations and (c) what barriers to implementation are currently seen (see Table 1). The interview guide was developed by the project team, guided by a review of the literature on palliative care and vascular surgery. It was validated during the first interview by using communicative validation.

**Table 1:** Interview guide

Category	Question	Follow-up questions
Importance of palliative care in vascular surgery	Do you agree with William Campbell’s statement that more palliative care should be implemented into vascular surgery?	• Could you briefly explain your answer? • What do you think such an improvement could or should look like?
Identification of palliative care patients in the vascular surgery setting	Which patient groups or “diagnoses” have a severely limited prognosis and require special support?	• For which of your typical patient groups or typical constellations of medical problems/diagnoses would you consider a prognosis of less than 12 months to be very likely? • For which patient groups would you consider supplementary palliative care to be useful, regardless of the specific vascular surgery treatment? • Do you have a tool to identify these patients with increased needs? Or do you use your intention more for this purpose?
Contents of palliative care treatment	In your opinion, what could or should such supplementary palliative care include?	• Symptom control, therapy goal, planning of post-stationary treatment • What would you hope the integration of palliative care would achieve? What goals would you demand?
Barriers to implementation	What barriers do you see in actual clinical care if palliative care is to be included?	• On the part of vascular surgery? • On the part of (specialized) palliative care?
Advance care planning	Would advance care planning be useful for palliative vascular surgery patients?	• For which situations? • Which aspects of advance care planning should be addressed or handled by palliative care team and which by vascular surgery?
Relatives	What role do relatives play in the care of seriously ill vascular patients?	• What are common problems faced by relatives of patients with vascular surgery problems in the advanced stage of the disease? • What kind of support should be offered to relatives?
Aspects not yet addressed, but subjectively relevant	In your opinion, are there any other important aspects of the interaction between palliative care and vascular surgery that we have not yet discussed?	

### Participants

Potential participants were selected from the regional and national network of the local university vascular surgery department. Inclusion criteria were: (1) currently working in vascular surgery, (2) more than 5 years of experience in vascular surgery (specialist/expert status), (3) responsible for independent therapy decisions, and (4) sufficient language skills (German language level ≥ B2).

### Data collection

The data were collected between September and November 2022 by the first and last authors (MR and RM). Both authors have many years of experience in specialized palliative care. The interviews were conducted via a protected video conferencing platform (red connect^®^).

### Analysis

The audio tracks of the interviews were recorded with the freeware Audacity, transcribed verbatim using MAXQDA^®^, and analyzed by MR and RM using the rule-based method of qualitative content analysis according to Kuckartz [6].

## Results

Thirty-seven physicians from all over Germany were invited. 11 physicians took part in the interview. Our interview partners were three women and eight men between 42 and 63 years of age. Six of them (54%) worked at a university hospital. None of the participants had acquired an additional qualification in palliative care at the time the interview was conducted.

The average interview length was 36 min (range: 21–53 min). Purposeful sampling and iterative data collection and analysis allowed us to assume theoretical saturation as early as interview 10. A total of 609 relevant text passages were identified and coded into seven main categories and 43 subcategories (Table 2).

**Table 2:** Categories and subcategories

Main category	Subcategory
1) Status quo of palliative care in vascular surgery	1. Currently still insufficient awareness of the necessity of palliative care content in vascular surgery 2. At the same time, increasing awareness of the necessity and growing importance of palliative care in vascular surgery 3. Cooperation with specialized palliative care already in some cases 4. Risk and incentives for overtreatment 5. Time for decision-making often limited 6. Lack of time resources 7. Numerous difficulties with regard to advance care planning 8. Inadequate knowledge of palliative care procedures among vascular surgery staff 9. Care of palliative vascular patients as a burden for vascular surgery staff 10. Inadequate post-inpatient care 11. Insufficient symptom control 12. Hospital often the place of death
2) Palliative vascular surgery patients	1. Disease 2. Relevant, frequent concomitant diseases 3. Multimorbidity and frailty 4. Decision of the patient against further surgical therapy 5. Technical limitations
3) Palliative care symptoms requiring treatment	
4) Identification of vascular surgery palliative care patients requiring palliative care support	1. Intuition 2. Relationship to patient 3. Discussion with the patient
5) Desired effects of possible palliative care	1. Improved symptom control 2. Enabling best supportive care 3. Holistic care 4. Appropriate care of the dying 5. Addition to geriatrics closes a gap in care 6. Support in determining therapy goals 7. Transfer of patients 8. Support in organizing appropriate post-inpatient care 9. Psychological relief provided for the vascular surgeon 10. Optimization and support for advance care planning
6) Barriers to implementation	1. Not always sufficient acceptance of a “palliative situation” 2. Lack of motivation for palliative care on the part of vascular surgeon 3. Inadequate palliative training 4. Fear of legal consequences 5. Monetary aspects 6. Insufficient capacity in specialized palliative care 7. Prognosis uncertainty 8. Palliative care is equated with dying 9. No time for palliative care
7) Relatives	1. Key role 2. Existence of an independent need for support due to multiple burdens 3. Lack of supply for relatives 4. Involvement in decision-making processes

The seven main statements in the interviews (corresponding to the seven main categories) are:

1) Currently there are numerous challenges and problems concerning the care of patients with a limited prognosis in vascular surgery (category “status quo of palliative care in vascular surgery”).

2) Palliative vascular surgery patients are considered to be a very heterogeneous group with different main and typically also concomitant diseases. All of those diagnosis are relevant to the prognosis: “We are as old as our vessels are”. On the basis of specific characteristics, participants describe situations which, in their opinion, are associated with a limited life expectancy and may therefore require palliative care. This group of patients is called “palliative vascular surgery patient” (category “palliative vascular surgery patients”).

3) A wide range of symptoms relevant to palliative care are mentioned, which should play a key role in further care (category “palliative care symptoms requiring treatment”).

4) A requirement for optimal care is the timely identification of palliative patients with a (main) vascular surgery diagnosis (category “identification of vascular surgery palliative care patients”).

5) Numerous aspects could improve palliative care for vascular surgery patients. The desired effects of intensifying palliative care affect a variety of dimensions (category “desired effects of possible palliative care”).

6) Although vascular surgeons expect the integration of palliative care and palliative content to improve patient care, a number of barriers for integration in everyday clinical practice are also described (category “barriers to implementation”).

7) Vascular surgeons attribute an important role to relatives in the palliative care of vascular surgery patients. This is expressed in various aspects that should be taken into account for a specific improvement in the care of relatives (category “relatives”).

The detailed statements of the interviewed experts within the seven main categories outlined above are presented in the following (with the subcategory number indicated at the beginning of the corresponding text passages; see Table 2):

### 1) Status quo of palliative care in vascular surgery

(1.1) The poor prognosis of many health problems related to vascular surgery is often not sufficiently recognized. On the other hand, vascular surgery problems and existing concomitant diseases can effect quality of life negatively. If one takes these aspects into careful consideration, the need for support from palliative care must be eventually recognized. (1.2) Awareness of palliative care as an important aspect of patient care has increased in recent years. It has now been recognized that conservative and, if necessary, internal treatment of vascular patients plays an equally important role when compared to surgical care. Palliative care should be expanded in vascular surgery in the future. (1.3) To some extent, cooperation with elements of specialized palliative care already exists. The main reason for this interaction is usually a request for transfer to the palliative care ward. Palliative care consultation services are also regularly required when the request for transfer to the palliative care ward cannot be satisfied. However, palliative care involvement often takes place rather late in the course of the disease. (1.4) With regard to end-of-life care, the risk of possible overtreatment is mentioned, outlining two main causative incentives: (a) Indication for treatment has shifted due to improving endovascular interventional techniques. Due to this, different interventions can be offered to a wider spectrum of patients. (b) Monetary considerations and financial incentives often push physicians towards treatments that are too extensive. (1.5) A characteristic feature of potential end-of-life situations in the context of vascular surgery is the often relatively narrow decision-making corridors in terms of time. (1.6) Palliative care or appropriate communication with the patient regarding the treatment planning usually fails because there is not enough time available. (1.7) Forms of advance planning are considered a particularly important aspect in the care. However, this is not sufficiently taken into account in everyday clinical practice (lack of discussions with patients, ultimately leading to avoidable re-admissions). (1.8) Physicians working in vascular surgery do not feel sufficiently qualified to care for palliative vascular surgery patients. There is a lack of (basic) knowledge about the aims and possibilities of palliative care. (1.9) The care of palliative vascular surgery patients and their specific challenges are considered to be very stressful. Therefore, vascular surgeons feel that suitable forms of psychological relief for staff are necessary. (1.10) Post-inpatient care for vascular surgery patients is described as inadequate and often unstructured. Many patients require extensive care, which is often difficult to provide at home or in residential facilities. Insufficient communication with outpatient care providers makes sufficient care even more difficult. (1.11) Palliative vascular surgery patients suffer from various symptoms (see Table 4), but these symptoms are not treated in the best possible way. Compared to the strategies for oncology patients’ care is inadequate. (1.12) Many vascular patients die in hospital.

### 2) Palliative vascular surgery patients

The findings are summarised in Table 3.

**Table 3:** Palliative vascular surgery patient

Diseases	• advanced peripheral arterial disease (Rutherford category 4–6), with/without necrosis or chronic wounds • impending first or repeated amputation • infected vascular prosthesis • “Complex aortic cases”, advanced aortic aneurysms, aortic rupture • revisions to prostheses, with “significant complications” such as intestinal ischemia, gut resections, dialysis • carotid stenosis (at least if concomitant diseases are present) • recurrent “shunt problems” • many previous vascular surgery procedures
Relevant and frequent concomitant diseases	• Numerous relevant serious concomitant diseases are mentioned that complicate vascular medical treatment. We often find ourselves at the interface with geriatrics • Internal diseases: terminal renal insufficiency or need for dialysis, diabetes mellitus with end organ damage, severe obesity, sarcopenia, cardiac insufficiency • Vascular diseases: severe chronic heart disease, strokes • advanced dementia • (very) high age (not a disease per se, but a condition)
Multimorbidity and frailty	It is not uncommon for several of these concomitant diseases to complicate vascular surgical care (multimorbidity). In addition to this, vascular surgery patients are often characterized by severe age-related limitations (frailty). Comorbidities often have a significant impact on the patients’ treatment in that they can limit the ability to undergo surgery or anesthesia.
Decision of the patient against further surgical therapy	In addition to medical causes for treatment limitations, patients themselves also request treatment limitations.
Technical limit	More and more frequently, no further surgery can be offered for technical reasons because all options have been exhausted.

### 3. Palliative care symptoms requiring treatment

The responses are shown in Table 4.

**Table 4:** Palliative care symptoms requiring treatment

physical problems	• pain and ischemia pain • painful changing of a wound dressing • bleeding • wound management of complex and chronic wounds, also associated with bleeding, odor formation and secretion formation • sepsis • pruritus • dyspnea
other dimensions	• psychological support • social support • spiritual support • post-inpatient care • nutritional therapy in the palliative setting

### 4) Identification of vascular surgery palliative care patients requiring palliative care support

(4.1) There are no validated tools used to identify patients requiring palliative care support, at least not regularly. At present, identification is essentially based on the intuition of the attending vascular surgeon (“gut feeling”). (4.2) The often long-standing relationship with the patient is seen as an opportunity for good therapy stratification. (4.3) The individual approach requires a shared discussion of the medical situation and decision making between the experienced physican and patient.

### 5) Desired effects of possible palliative care

(5.1) Optimized pain therapy is a major concern. However, improved, comprehensive symptom control that goes beyond pain management and offers the patient extensive relief from suffering is an important objective. (5.2) Patients who do not receive a surgical procedure should still be offered appropriate and dignified care. (5.3) Non-physical symptoms should also be treated appropriately. Individual and holistic care, i.e., “human medicine” with the inclusion of social and spiritual dimensions, is seen as desirable. (5.4) Improved palliative care also includes a special focus on the holistic care of the dying, which is currently considered to be in urgent need of improvement. (5.5) Interdisciplinary teamwork with palliative care opens up a range of care options for patients with complex conditions who do not qualify for geriatric treatment. This could close an existing gap. (5.6) Interaction with palliative care is perceived as an opportunity to discuss difficult questions regarding further therapy and for determining a realistic treatment goal. (5.7) Vascular surgeons are explicitly seeking palliative care to transfer such patients to a palliative ward. (5.8) Optimizing or supporting the organization of appropriate post-inpatient care is considered important. One aim would also be to enable patients to remain at home if they wish and to avoid further hospitalization. (5.9) The integration of specialized palliative care would also contribute to the personal psychological relief of the vascular surgeon. Relief would be gained through the awareness that the patient is well cared for despite the “lack” of vascular surgery intervention. (5.10) Appropriate planning could ultimately avoid a lot of pointless procedures but is often missing in daily clinical practice. Anticipatory care planning in the context of a specific intervention is seen as a central task. For some palliative situations (such as a large abdominal aortic aneurysm), there are often few or no relevant symptoms that require (specialized) palliative care at that point. However, appropriate advance care planning (ACP) may be considered for future scenarios.

### 6) Barriers to implementation

(6.1) Often, a “palliative situation” cannot be accepted as such by the surgeon. This is particularly true if the patient’s current poor condition is seen as a complication of the doctor’s “own” surgery. In addition, some vascular surgeons believe that symptom control based solely on medication is inferior to surgery in terms of quality of life. (6.2) Integration of palliative care is also not always perceived as a personal responsibility of vascular surgeons. (6.3) Vascular surgeons’ own training regarding palliative care is valued as inadequate to enable them to care appropriately for their palliative patients. (6.4) There is uncertainty among vascular surgeons as to which end-of-life decisions are legally permissible. There is a concern about the legal consequences of not always offering every medically and technically possible treatment. (6.5) Financial considerations and constraints are cited as major barriers to more palliative care. There is great economic pressure to perform many surgeries. Conservative and palliative care worsens key performance indicators and are not reflected in funding options. (6.6) The capacities available for specialized palliative care (for vascular surgery) are perceived as too limited. This applies to the capacity to transfer patients to the palliative care ward and to the care capacities in outpatient (specialized) palliative care teams. (6.7) It is often difficult to assess the prognosis of the individual patient at a certain point in time. This often complicates the acceptance of palliative approaches. (6.8) Many patients and vascular surgeons “only” associate the term palliative care with the medical treatment of the dying. This barrier must be overcome with appropriate education if greater implementation is to be achieved. (6.9) Vascular surgeons see the lack of time as an insurmountable barrier to their own palliative care activities (including appropriate training).

### 7) Relatives

(7.1) Family caregivers play a crucial role in palliative care. They are extremely important for the care of patients at home, especially due to the high proportion of elderly people in the group of patients. (7.2) Due to the many burdens and challenges resulting from their care for the patient, there is a need for support specifically geared towards the patient’s family members. Furthermore, relatives have a special need to talk about their experience. By taking the special needs of relatives into account, care can often be improved. (7.3) In practice, despite knowledge of the aforementioned needs, there is a relevant lack of care for relatives. (7.4) The involvement of relatives in key decision-making processes is considered to be very important. However, this can also result in burdensome choices. This applies not only to questions of care, but also to difficult decisions about treatment goals.

## Discussion

The scant literature available to date on palliative care for vascular surgery patients is based primarily on clinical patient data. The results of our study, which we were able to extract from interviews with experienced vascular surgeons and their insights into everyday clinical practice, confirm the conclusions drawn in the clinical studies and supplement them with further aspects, which together appear necessary for improved care of this patient group in the future.

### Integration

Our survey shows diagnoses and medical circumstances that the experts consider to be palliative situations. The integration of palliative care and structures has essentially only been achieved in oncology [2], but not in other areas of medicine [7, 8]. There is a lack of palliative care particularly in vascular surgery [9–13]. Even though vascular surgeons are gradually becoming more cognisant of the “palliative” situations that arise in vascular surgery patients, awareness of and access to palliative care remains inadequate in clinical practice (insufficient scope of offers, too late). This results in a deficiency in the provision of care for a highly vulnerable patient group with a significantly limited lifespan, consequently leading to an unnecessarily diminished quality of life. These phenomena, which are predominantly supported by clinical data and documented in the literature [14–20], have been substantiated by the statements of the interviewed vascular surgery experts.

In the extant literature, the question of which specific (palliative care) interventions should be used for vascular surgery patients remains largely unresolved [21]. Initial studies suggest that the early [22] involvement of specialized palliative care in the form of consultations with specialized palliative care staff and the application of advance care planning [4, 10, 11] could make an important contribution to more comfort care [4, 15]. (Specialized) palliative care is a valuable resource for vascular surgeons. It can provide additional time, better symptom control, support in difficult conversations, and a structured discussion of medical and ethical questions [4, 15, 23]. It may also help to reduce the fear of legal consequences when refraining from maximum therapy [14].

The identification of palliative patients is essentially based on intuition, partly based on the aforementioned diagnoses and accompanying circumstances. Vascular surgeons find it difficult to determine an individual prognosis or to define specific points in time from which palliative care should be involved, which is in accordance with the literature [24, 25].

Intuition in recognizing palliative situations (e.g., in the form of the “surprise question”) has also been shown to be a possible option in the context of oncology and other diseases [18, 26, 27]. The significance for vascular surgery remains uncertain, although many “palliative diagnoses” have a life expectancy of less than one year.

In the course of the interview, participants were invited to name a number of vascular surgical diseases, as well as the accompanying circumstances or characteristics of the treatment situation in which they would expect a “palliative” situation. Many advanced diseases from the spectrum of vascular surgery, the vascular surgical diagnosis itself or accompanying severe comorbidities have a (very) poor prognosis for survival [28–30]. This is still true for many patients, even after they have undergone vascular surgery [31], which may be partly due to these comorbidities [32–35]. Palliative care and surgical intervention are not necessarily mutually exclusive; however, the risks, opportunities and quality of life with and without surgery must be consciously weighed for the patient [36]. In contradistinction to the UK [37, 38], for example, there are no guidelines in Germany that recommend the standard inclusion of palliative care based solely on a potentially limited prognosis for a specific diagnosis. However, this could be helpful. At the same time, identification by medical staff alone has been shown to be insufficient [39]. Considerations in the direction of standardised screenings and inclusion of the patient perspective could represent useful further developments [40, 41]. Whether the tools tested in oncology are also useful for the typical palliative vascular surgery patient remains to be investigated due to the characteristics of vascular patients [42].

### Barriers

Despite the high requirements, palliative care is integrated much less frequently than in oncology [43] and to an insufficient extent [12], 14– [10, 20, 44]. Numerous barriers were identified; knowledge of palliative care issues appears to be important for more effective implementation. Many of the barriers and difficulties named by vascular surgeons that would prevent the timely integration of palliative content are known (in this or a very similar manner) from the oncological setting [45–47], where this issue has been addressed in detail for more than a decade. It should be investigated to what extent the experience gained in overcoming these barriers and the concepts developed can also be applied, at least in part, to the vascular surgery situation. The training of vascular surgeons in palliative care is of potential central importance. In everyday clinical practice, the structured recording and documentation of the current medical situation, the patient’s individual care preferences and a comparison with the specific medical findings, as well as the joint decision-making at the end of the process, can be beneficial [15]. 

### Symptom management

Due to the diseases and symptoms in vascular medicine, a high demand for palliative care can be assumed. This could be substantiated by our survey. The symptoms and needs described by the experts interviewed extend beyond the purely physical dimension, encompassing psychological, social and spiritual aspects, as addressed in the holistic approach of palliative care. Many of the aspects mentioned are again comparable to oncological palliative care patients and are well-known aspects of palliative care [48–50]. The trajectories of vascular surgery and oncology patients typically differ, so that specific ways of implementing palliative care must also be evaluated and discussed [51].

### Definition of the term “palliative care”

The patients’ problems to be addressed described in the interviews are an (unsorted) mixture of basic and specialized palliative care. Further differentiation of tasks and their localization in one of the sectors is an important requirement for the expansion of palliative care content. Palliative care in Germany is provided by two sectors: basic palliative care, which is usually provided by the general practitioners and consultants, and supplementary specialized palliative care. Specialized palliative care is provided by a multidisciplinary team that is specifically qualified in palliative care and has its professional focus here [2, 52, 53]. 

Furthermore, the interviewees subsume all the elements discussed under the term palliative care, without differentiating them clearly. The deficiencies and problems highlighted in the interviews relate to a variety of elements of palliative care, which can be offered at different times during the illness and with different intentions. The terms “palliative care”, “end of life care”, “surgical palliative care” and “terminal care” therefore require a structured definition for clear language [15, 44, 54]. The lack of conceptual clarity itself hinders access to palliative care for doctors, patients and relatives [15, 54]. 

The existing (inter)national guidelines were not addressed at all by the interviewees, and the terminologies were not distinguished independently. This underlines the self-assessment of the interviewees of insufficient knowledge of palliative content, and thus represents approaches for improvement, for example through targeted training [15].

The findings of this study indicate that vascular surgical palliative patients may represent a distinct group, thus necessitating the development of a tailored concept and translation of fundamental palliative care concepts to the specific needs of this group. This approach should incorporate the various elements discussed above, in the sense of “vascular surgical palliative care”, namely “the treatment of suffering and the promotion of quality of life for seriously ill or dying patients” with a leading vascular surgical diagnosis [15, 55]. 

### Relatives

The special role of relatives in palliative care, specific problem areas, particular burdens and needs and a requirement for support that is independent of the patient were identified in the interviews. In addition to specialist questions, these are numerous aspects that have already been recognized as desiderata in the oncological setting [56, 57]. These findings from oncology could also be examined to determine whether and to what extent they are transferable to the situation in vascular surgery.

### Limitations and further research

This study is important in that it opens up a new area of investigation for palliative care research. However, certain limitations must be acknowledged. Because of the small sample size of this study, it is not possible to generalize the results. In addition, the inclusion criteria focused exclusively on the medical perspective. In principle, expert interviews have the potential risk of answers being given for reasons of social desirability. It was not required that the experts themselves had completed palliative care training. As a result, relevant aspects may not have been considered because they were not perceived as important. Above all, however, the present study has succeeded in providing an unfiltered perspective on the current care situation. Further studies could re-evaluate the consistency of the identified results with a larger group of participants. Other disciplines (e.g., nursing staff, social workers) could also be included, which would be even more appropriate with regard to the multi-professional nature of palliative care. Finally, the patients and relatives themselves should also be surveyed about their subjectively perceived need for support.

## Conclusions

This research contributes to a better understanding of what needs to be implemented to improve palliative care for vascular surgery palliative care patients. Our findings provide a detailed and rich description of the experiences of vascular specialists with regard to necessary and missing aspects of palliative care for vascular surgery patients with palliative conditions. The statements of the interviewed experts confirm the challenges, deficits and development needs outlined in the scarce literature on the interface between vascular surgery and palliative care, so that a better quality of life can be achieved for palliative patients with leading vascular surgical diagnoses. Central aspects to be considered include suitable training of vascular surgeons for a comprehensive understanding of palliative care and the skills used in this setting (such as multidimensional symptom control, legal and ethical issues in life-threatening diseases), as well as forms of advance care planning that are suitable for this specific patient group. Written standards defining the situations in which palliative care should routinely be offered (as described in the “palliative vascular surgery patient group”) could also be a helpful step towards implementing palliative care in a more needs-oriented way. Our findings also show which questions future interdisciplinary research projects could address.

## Data Availability

No datasets were generated or analysed during the current study.

## Abbreviations

ACP: advance care planning
